# Microphotonic needle for minimally invasive endoscopic imaging with sub-cellular resolution

**DOI:** 10.1038/s41598-018-29090-6

**Published:** 2018-07-17

**Authors:** Mohammad Amin Tadayon, Ina Pavlova, Kelly Marie Martyniuk, Aseema Mohanty, Samantha Pamela Roberts, Felippe Barbosa, Christine Ann Denny, Michal Lipson

**Affiliations:** 1 Department of Electrical Engineering, Columbia University, New York, NY USA; 2 Department of Psychiatry, Columbia University, New York, NY USA; 3 Department of Physics, University of Campinas, Campinas, SP Brazil

## Abstract

Ultra-compact micro-optical elements for endoscopic instruments and miniaturized microscopes allow for non-invasive and non-destructive examination of microstructures and tissues. With sub-cellular level resolution such instruments could provide immediate diagnosis that is virtually consistent with a histologic diagnosis enabling for example to differentiate the boundaries between malignant and benign tissue. Such instruments are now being developed at a rapid rate; however, current manufacturing technologies limit the instruments to very large sizes, well beyond the sub-mm sizes required in order to ensure minimal tissue damage. We show here a platform based on planar microfabrication and soft lithography that overcomes the limitation of current optical elements enabling single cell resolution. We show the ability to resolve lithographic features that are as small as 2 μm using probes with a cross section that is only 100 microns in size. We also show the ability to image individual activated neural cells in brain slices via our fabricated probe.

## Introduction

The size of endoscopic instruments with high resolution at the sub-cellular level, is limited by the current micro-optical elements, which in turn are constrained to large sizes due to their manufacturing process. Optical fiber bundles^1,2^, miniaturized lenses^2,3^, and gradient index (GRIN) lenses^4^ have been applied for endoscopic imaging. Optical fiber bundles have been utilized for epifluorescence *in vivo* calcium imaging^5^, confocal imaging^6^, and two photon imaging^7^. However, they suffer from lack of resolution, determined by size of the fiber^6^. Miniaturized lenses have been applied for endomicroscopes^2^, and confocal imaging^8^. However, since miniaturized lenses are achieved via micromanipulation and polishing^9^, their size is usually limited to millimeter scale. GRIN lenses have been utilized to image brain tissue using single^10–13^ or two-photon^13^ imaging techniques. However, they have a typical diameter of approximately 0.5–1 mm. These lenses have successfully been utilized for optical coherence tomography imaging of a coronary artery^14^ and esophagus^15^. In neuroscience studies, GRIN lenses have successfully imaged deep layers of the brain^10^ and have enabled the study of deep brain areas including hippocampal CA1 for memory encoding^16^ and hypothalamic network for appetitive behaviors^17^. However, GRIN lenses suffer from resolution and cross sectional area tradeoff. This tradeoff is fundamental and originates from the difficulty of forming the necessary strong gradient of the refractive index (determined by the glass doping concentration) within a small cross sectional area. Due to the difficulty in achieving such a gradient, in order to ensure focusing, these GRIN lenses typically have large cross-sectional dimensions, between 0.5 mm and 1 mm in diameter.

**Figure 1:**
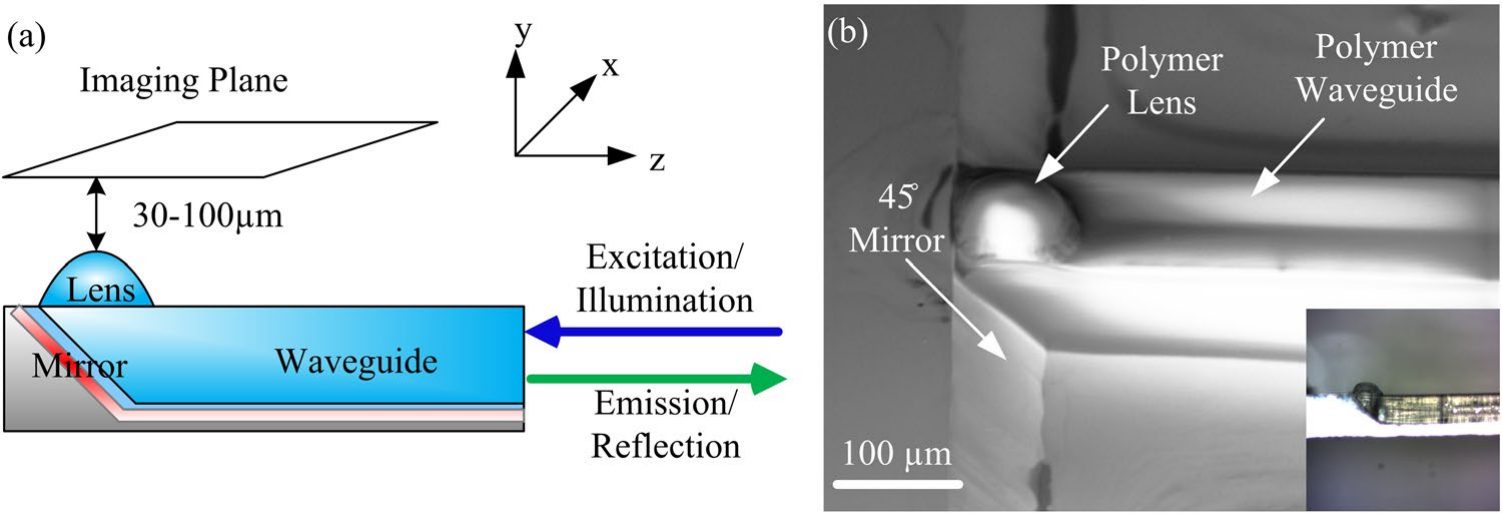
(**a**) Schematic of the probe consisting of a polymeric waveguide, aluminum mirror and polymeric micro-lens. (**b**) SEM image of a fabricated probe with 80 μm × 100 μm in cross section integrated with a micro-lens with an approximate curvature radius of 45 µm; Inset shows a microscope image of the probe with the substrate thinned down to less than 50 µm.

We show here a platform based on planar microfabrication that overcomes the limitation of current optical elements enabling single cell resolution using probes with a cross section that is only 100 microns in size. The probe consists of a polymeric waveguide monolithically integrated with a high Numerical Aperture (NA) microlens composed of a polymer molded into a semi-sphere (Fig. 1a). These lenses are shaped using lithographic processes. Since the lensing effect in these micro-lenses is based on the shape and refractive index of the lens, they can have a diameter that is only tens of microns. This new micro-fabricated probe technology enables one to achieve high resolution (i.e. high NA) while keeping the cross section of the probe small, between 500 microns and down to only a few microns in diameter. This is in contrast to the traditional GRIN lens with a large millimeter size cross section.

## Methods

**Figure 2:**
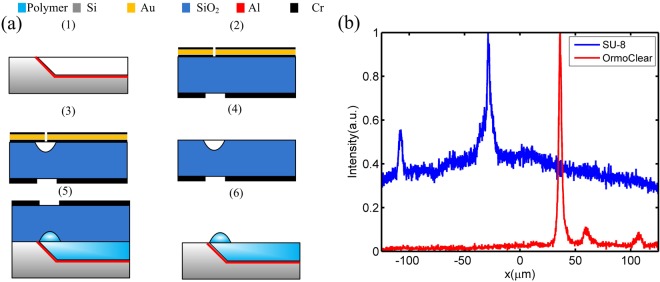
(**a**) Fabrication process of the probe. (**b**) Fluorescence imaging of a single 6 μm fluorescent bead (with peak emission at 520 nm), via a probe fabricated using low absorption polymer Ormoclear®FX and for comparison via a probe fabricated using SU8. Both of these image profiles were taken using a 50× objective with an NA of 0.55 and a monochromatic scientific CMOS camera. The images are normalized to their respective peak intensities. One can see that the SNR level for the low absorption polymer is much higher due to its low background fluorescent emission.

The platform leverages standard silicon manufacturing for massive scale manufacturing and relies on soft lithography for the fabrication of 3D photonic structures. (Fig. 2a). The probe substrate is defined using wet etching of silicon (Fig. 2a, step 1) utilizing a silicon dioxide hard-mask patterned with a 45° angle with respect to the silicon substrate edge^18,19^. Aluminum layer acting as a mirror is deposited on the silicon substrate. The lens mold on a fused silica substrate is fabricated using circular patterns defined by a mask^20,21^ and wet etching using a HF:DI solution to form the semi-spheres concave surfaces (Fig. 2a, steps 2–4). The mold is aligned to the silicon substrate and OrmoClear®FX (micro resist technology GmbH) is flown into the waveguide mask-and-lens mold by decreasing its viscosity and using capillary forces via careful temperature control of the substrate (Fig. 2a, step 5). The final step consists of exposing the probe to UV light for patterning and curing the polymer and, finally, releasing the probe (Fig. 2a, step 6). Following the fabrication of the probe the back side of the silicon substrate can be etched down to a few tens of microns using reactive ion etching (Fig. 1b inset). The SEM image of the resulting structure is shown in Fig. 1b. More details on the fabrication process can be found in Supplementary 1.

The unique microfabrication methods of our process, in contrast to traditional manufacturing processes, enables one to tailor the resolution of the probe, while enabling millimeters long probes. The resolution can be tailored by designing the shape of the lens, defined by the etching time and the mask shape used in the fabrication of the lens mold^22,23^. The length of the probe *l* is given by: *l* = *2nfw/D*, where *D*, *w*, *n*, *f*, are the field of view diameter, waveguide width, waveguide refractive index, focal distance, respectively. Assuming *w* = 100 μm, *D* = 50 μm, n = 1.56, and f = 800 μm, a probe can be several millimeters long and still maintain cellular resolution (more details presented in Supplementary 4).

In order to overcome the auto-fluorescence which has limited polymer photonics for fluorescence imaging, we minimize the energy transfer from the excitation light source to the polymer by engineering a hybrid polymer based device with ultra-low absorption in the whole visible range^24^. We use Ormoclear®FX – a polymer developed by micro-resist GMBH with an absorption of less than 1% between 350 nm and 800 nm. We show the ability to image a single fluorescent bead with high signal to noise ratio (SNR) overcoming the traditional source of noise due to the high auto-fluorescence in polymers^25^. Figure 2a shows the fluorescence imaging of a single 6 μm fluorescent bead (with peak emission at 520 nm), via a probe fabricated using low absorption polymer Ormoclear®FX and, for comparison, via a probe fabricated using SU8. SU8 is one of the standard polymer for fabrication of the polymer waveguides. Both of these profiles were taken using a 50× objective with an NA of 0.55 (Mitutoyo M Plan Apo 50×) and a monochromatic Scientific CMOS camera (ORCA flash 4.0 LT Hamamatsu Photonics). One can see that the SNR level for the low absorption polymer is much higher due to its low background fluorescent emission.

## Results

**Figure 3:**
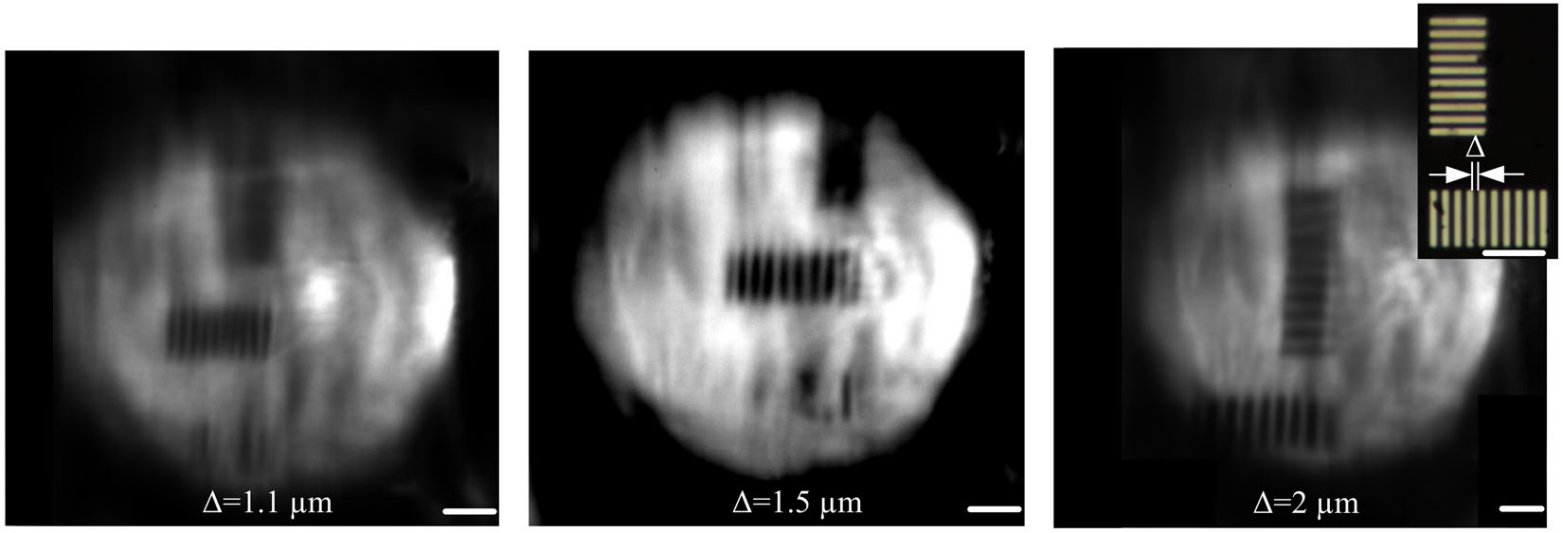
Images of lithographically defined metallic lines spaced by Δ which varies between 1.1 μm and 2 μm through a fabricated probe. The probe used was 80 μm × 100 μm in cross section, was integrated with a micro-lens with an approximate radius of curvature of 45 µm and was fabricated using a polymer with an index of 1.56. We measure a lateral resolution of 1.1 µm along one direction and less than 2 µm along its perpendicular direction. All the scale bars are 10 μm.

We show the ability of the probe to resolve lithographic features that are as small as 2 μm consistent with our theoretical predictions based on the measured profile of the lens and the calculation of the aberration. In order to determine the resolution of the probe, we imaged lithographically defined metallic lines spaced by Δ which varies between 1.1 μm and 2 μm μm through a fabricated probe. The probe used was 80 μm × 100 μm in cross section and was integrated with a micro-lens with an approximate curvature radius of 45 µm. The entire probe structure was fabricated using a polymer with an index of 1.56. The metallic lines were defined using electron beam lithographic patterning of an aluminum film deposited on a fused silica substrate. White light is sent through the probe for illumination and the reflected light off the object through the probe is then collected through a 50× objective with a NA of 0.55 and imaged onto a scientific CMOS camera. The working distance between the object and the lens is about 100 μm. We measure a lateral resolution of 1.1 µm along one direction and less than 2 µm along its perpendicular direction (see Fig. 3). The difference in resolution between the lateral and axial axis is due to the difference in the waveguide geometry with 80 µm thickness and 100 µm width. We performed most of the experiments in air but device can be immersed in the water as well.

**Figure 4:**
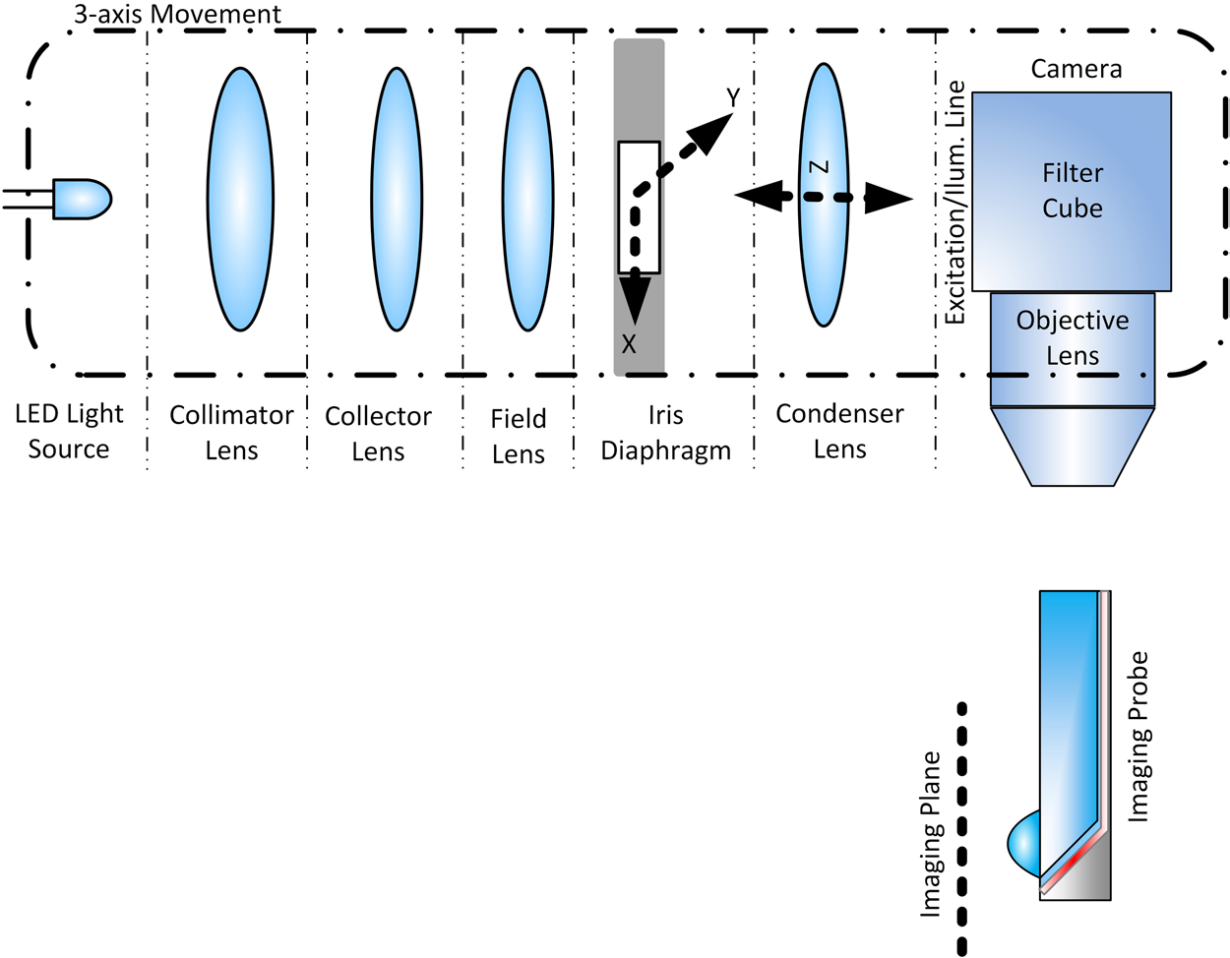
Optical imaging setup for illumination and imaging from the endoscopic probe.

The imaging set-up shown in Fig. 4 has been utilized to characterize the probe. To illuminate the imaging target and reconstruct the image formed behind the lens, we use the free space imaging set-up located behind the waveguide. This setup can be replaced with any reflected light microscopy system. We use the Köhler illumination method for the illumination part of the imaging setup. For the case of the reflected light microscopy, this type of illumination provided a better contrast. The image replica of the iris diaphragm is on the image plane of the imaging setup. This is used to make the illuminating area as small as the waveguide area. The optimization of the illumination line helps to improve the contrast in the image, but a good quality image can be achieved even without optimization of the illumination line. We utilized a 50× objective with an NA of 0.55 (Mitutoyo M Plan Apo 50×) and a monochromatic Scientific CMOS camera (ORCA flash 4.0 LT Hamamatsu Photonics) for all of the imaging. For more details on the measurement set-up please see Supplementary 2.

**Figure 5:**
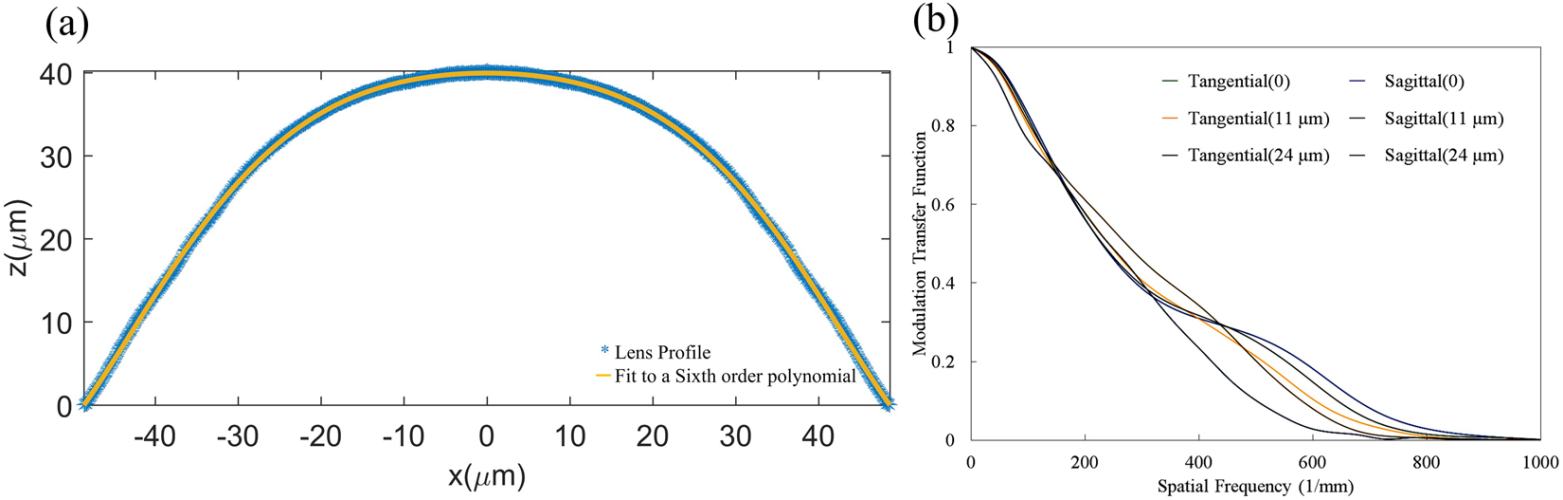
(**a**) The fabricated lens profile. Dots are extracted from the microscope image and solid line is a fit to a fourth order polynomial. (**b**) Calculated Modulated Transfer Function at the lens focal plane for a wavelength of 550 nm using the approximated fit profile at the focal plane of the lens using OpticStudio (Zemax LLC). One can see from the MTF of the lens that the cut-off resolution of our probe is about 700 lp/mm is in agreement with our experimental imaging results.

We quantify the monochromatic aberrations of the lens defined by the lens shape and show that although the lens shape induces some aberrations, the resolution remains well within the range necessary for imaging individual cells. In Fig. 5a, we show the lens profile extracted from the lens mold profile and the fit to a sixth order polynomial. Using this fit, we calculate the modulation transfer function (MTF) diagram at the focal plane of the lens for a wavelength of 550 nm across the lens field of view, using OpticStudio (Zemax, LLC) (see Fig. 5b). One can see from the MTF of the lens that the expected resolution of our probe is in agreement with our experimental imaging results. The cut-off resolution of the probe is about 730 lp/mm and the resolution of the probe based on the Rayleigh criterion (Airy disk radius) is 1.3–1.4 μm. Note that, in principle, using multi-lenses these aberrations could be alleviated in the future using the method presented in^26,27^.

**Figure 6:**
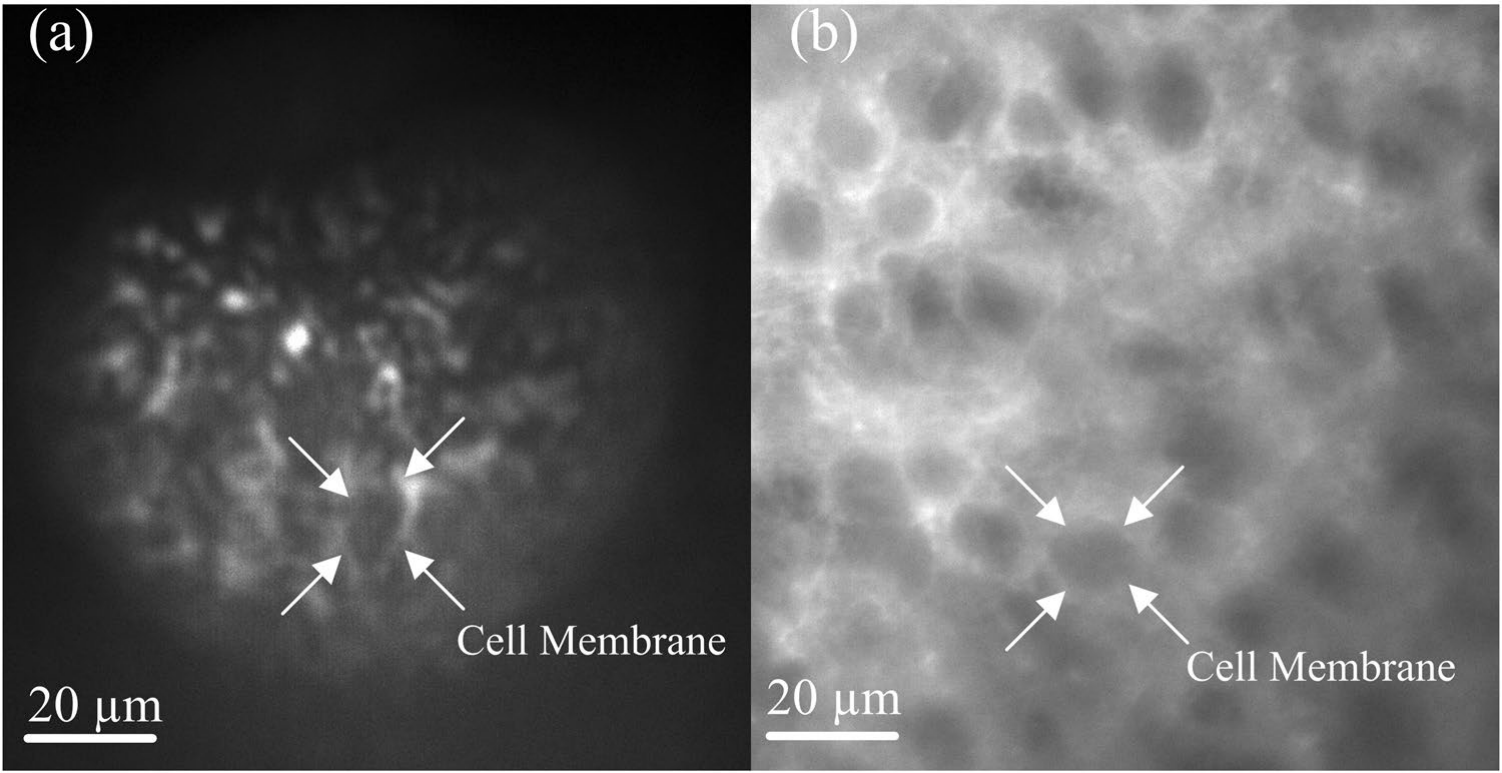
Wide-field microscopy of activated neural cells in brain slices of activated neural cells taken from the cerebral cortex region of an ArcCreERT2^34^ x ChR2- enhanced yellow fluorescent protein (EYFP)^29^ transgenic mouse^28^. This mouse line offers permanent membrane bound EYFP labeling of Arc^+^ neurons, which were activated during the learning of an experience^34^. (**a**) The image obtained using the fabricated microprobe the captured image shown shows differentiated neuron boundaries with a FOV of 65 μm. (**b**) The image collected using a standard bench top microscope.

As an example of an application of our platform, we show cellular level resolution by imaging activated neural cells in brain slices using wide-field microscopy via our fabricated probe. Wide field microscopy has been used for imaging neural dynamics in various brain structures^27,28^. Unlike conventional two-photon microscopy, in wide-field microscopy all pixels in an image are sampled simultaneously. Therefore, this method is advantageous over two-photon microscopy for high speed functional imaging. Figure 6a shows images of slices taken from the cerebral cortex region of an ArcCreERT2^29^ x ChR2- enhanced yellow fluorescent protein (EYFP)^30^ transgenic mouse obtained using the fabricated microprobe. This mouse line offers permanent membrane bound EYFP labeling of Arc^+^ neurons, which were activated during the learning experience^29^. The captured image shown in Fig. 6a clearly shows differentiated neuron boundaries with a FOV of 65 μm. More details on the preparation of slices can be found in Supplementary 3. For comparison we also show in Fig. 6b the image collected using a typical image of the cerebral cortex using the regular microscope (standard bench top microscope). Note that due to the tagging used here, only the cell membranes fluoresce while the encapsulated cells within the membranes appear darker in the image.

The same probe has been inserted in the mouse left visual cortex for optogenetic excitation of neurons and the insertion damage to the tissue can be seen in^31^. The probe can be inserted either with a thinned substrate or as a standalone polymeric lens waveguide. The polymeric waveguide-lens probe can be released from the substrate and inserted. The insertion force of the 100 μm flat probe to the brain cortex is about 0.5–0.6 mN^32^. The critical force for the buckling of the probe based on the Euler’s buckling equation is, F_critical_ = Kπ^2^ EI/L^2^, where K, E, I, and L are column effective length factor, modulus of elasticity, area moment of inertia, and length of the probe respectively^33^. Considering Fixed-Pinned or Fixed-Fixed boundary conditions (K = 2–4)^33^, modulus of elasticity of the polymer (E = 4 GPa), the probe length of 5 mm, and cross sectional area of 100 μm × 100 μm, critical force to buckle the probe is 25–50 mN. Therefore, the buckling force of the standalone polymeric probe is significantly larger than the insertion force to the brain cortex.

## Conclusion

The demonstrated platform allows for a completely new generation of microprobes to be used in imaging for a variety of applications. In neuroscience, this can enable the implementation of multiple techniques (such as wide field microscopy and multi-photon imaging) used today for imaging and excitation of deep brain tissues and, in contrast to the GRIN lenses used today, can be minimally invasive. In medical engineering, it could enable emerging applications that require cellular level resolution for diagnostics involving multiple modalities techniques emerging today such as optical coherence tomography, fluorescence (auto-fluorescence or via an injected dye) imaging, and spectroscopy of the cells. Most of these techniques are being introduced in endoscopic instruments that are several mm wide and, therefore, limited to applications such as colon studies where such large endoscopic tools can be inserted. For example, OCT can be used for confirming clear margins in cancer removal procedures and minimally invasive imaging for replacing biopsies. Note that although we have shown here a side imaging probe, using the same fabrication technique (based on aligning micro fabricated molds) one can realize a forward imaging probe with multiple embedded lenses in order to ensure minimal aberrations and maximize the field of view for a probe length of several millimeters long.

## Electronic supplementary material

Supplementary Material
