# Supplementary material for: Microphotonic needle for minimally invasive endoscopic imaging with sub-cellular resolution
Source: Sci Rep. 2018 Jul 17;8:10756. doi: 10.1038/s41598-018-29090-6 (PMC6050293; doi:10.1038/s41598-018-29090-6)
Supplement: Supplementary file 1 — Supplementary Material [file 41598_2018_29090_MOESM1_ESM.pdf]

Microphotonic needle for minimally invasive endoscopic imaging with sub-cellular resolution

Mohammad Amin Tadayon[1], Ina Pavlova[2], Kelly Marie Martyniuk[2], Aseema Mohanty[1], Samantha Pamela Roberts[1], Felippe Barbosa[3], Christine Ann Denny[2], Michal Lipson[1]

[1]Department of Electrical Engineering, Columbia University, New York, NY, USA. [2]Department of Psychiatry, Columbia University, New York, NY, USA. [3]Department of Physics, University of Campinas, Campinas, SP, Brazil.

Supplementary 1- Details of the Fabrication

We fabricate an OrmoClear®FX waveguide (cross section: 80 μm×100 μm) and lens with an approximate curvature radius of 45 μm on a silicon substrate. We create a mirror that deflects the light at the end of the waveguide by 90° upward through the lens using a 45° angle step (in silicon {111} plane) which is coated by 200 nm of Aluminum. First the p-type {100} silicon substrate is coated with 800 nm of silicon dioxide which works as a mask for later etching of silicon substrate. This silicon dioxide rectangular pattern mask is made with a 45° angle with respect to the substrate edge. The silicon area that is etched is first exposed to a solution of 25% tetramethylammonium hydroxide (TMAH) mixed with 10-50 ppm Trionx100 at 90°C [12-13]. After etching the silicon substrate, the silicon dioxide mask is removed and a 200 nm Aluminum layer forming the mirror is deposited on the silicon substrate. The lens mold and the waveguide mask are made on a fused silica substrate. The lens mold is made by defining circular patterns on 30 nm Cr/200 nm Au/1400 nm AZ 1512 film (hard mask) on the fused silica substrate [14-15]. Next, a solution of 49% Hydrofluoric Acid: Deionized Water (1:1) is used to make the lens mold with semispherical patterns of about 45 μm radius. After removal of the hard mask, the Chromium mask which has the waveguide pattern is aligned with the mold and defined on the back side of the same substrate. The lens mold is treated in fluoroctatrichlorosilane (FOTS) to avoid stiction of the polymer to the mold in the next steps. The lens mold and the silicon substrate are aligned with each other and held together with a holder. Then the fixed mold and substrate are left on the hotplate with OrmoClearFX. The temperature of the hotplate gradually increases from 45°C to 95°C in about 30 minutes. Because of the increase of the temperature, the viscosity of OrmoClear®FX gradually decreases, and the polymer starts to flow between the mold and substrate due to the capillary effect. To avoid the cracks and rupture of the waveguides after exposure, every 10-15 minutes the temperature of the hot plate is decreased by 10-15°C up to room temperature. Finally, the substrate and mold are exposed, the mold is released, and the OrmoClear®FX waveguide/lens is developed in the OrmoClear®FX developer.

Different steps of the fabrication process can easily be modified to achieve different probe sizes with different numerical apertures. Removing the mirror, the microlens can be moved to the front of the waveguide to make a forward looking probe instead of side looking. By applying polymers with different refractive index (1.33-1.7), different numerical apertures can be achieved. For the fluorescence imaging applications, the polymer with very low autofluorescence properties should be selected. The micro-lens profile and size can be modified by changing the etching time and

process of the lens mold. The cross section of the waveguide can easily modified by changing the width of the waveguide on the waveguide mask and depth of the etching in silicon etch.

Supplementary 2- Imaging set-up and imaging performance

As it can be seen in the Sup-Fig.1, we apply the critical illumination (focusing the laser light inside the waveguide) in case of the fluorescence imaging, which allows us to achieve a higher contrast image. The laser excitation light deliver to the set-up using an optical fiber. The optical fiber output is collimated using an optical fiber collimator. The excitation light can be moved and aligned to the waveguide center using a kinematic mount by changing the collimated light angle.

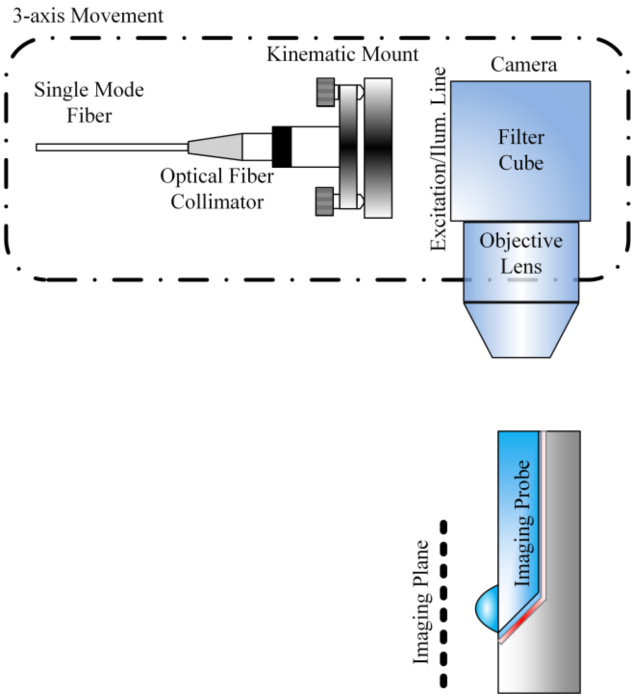

Supplementary Figure 1- Optical imaging setup for fluorescence imaging from the endoscopic probe.

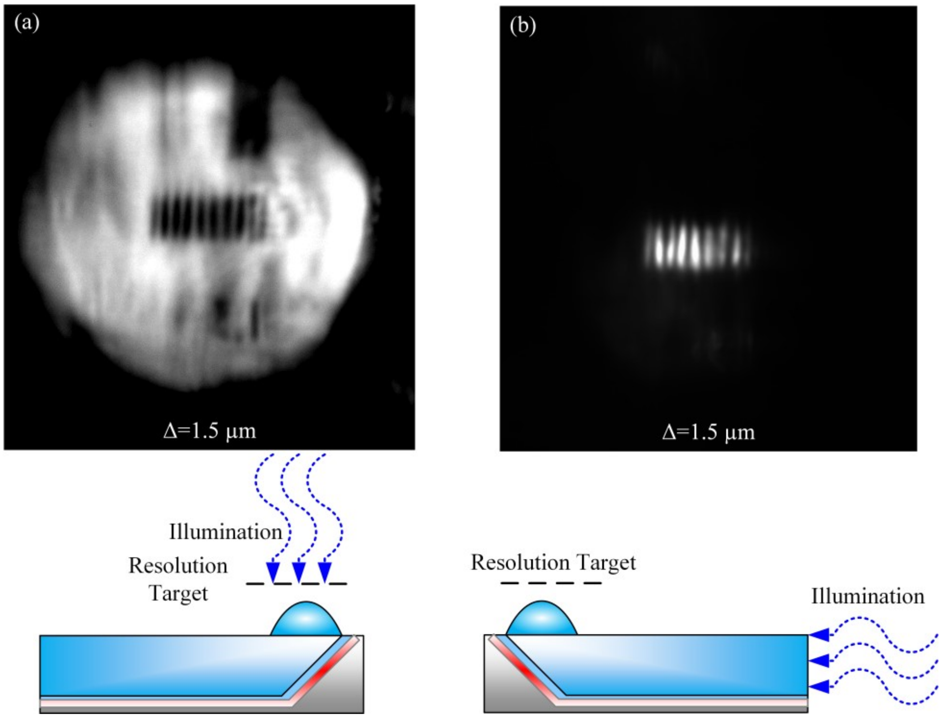

Supplementary Figure 2- imaging of the 1.5 µm target (a) with illumination from the object side (b) illumination from the waveguide side.

The imaging target can be illuminated either from the object side or through the waveguide. Sup-Fig.2a-b shows images produced from the same target using different illumination methods. Removing the background will help to increase the imaging contrast.

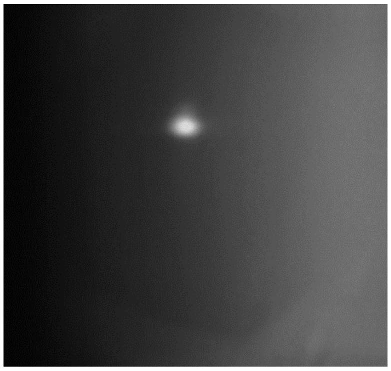

Supplementary Figure 3- Fluorescence imaging of a fluorescence bead using the endoscopic needle.

To test the probe's ability for fluorescent imaging, we also imaged 6 µm fluorescent microspheres (StarLight™ Calibration Slide) dyed with a single fluorophore with maximum excitation at 480 nm and maximum emission at 520 nm. The excitation has been done through the waveguide. We were able to image the microspheres (Sup-Fig.3) without any image processing.

Supplementary 3- eYFP transgenic mouse slice preparation

To drive the expression of reporter eYFP in active neurons, 8-week-old ArcCreERT2 x ChR2-eYFP mouse received an intraperitoneal injection 4-hydroxytamoxifen. Five days later, the mouse

was perfused with cold 1X phosphate-buffered saline (PBS) immediately followed by 4% paraformaldehyde (PFA). Next, the brains were quickly removed and submerged in 4% PFA overnight at 4C. The following day, the brains were transferred to 1X PBS and 100 um thick coronal tissue sections were cut on a Leica VT1200 vibratome. Free-floating sections were kept in 1X PBS with 0.01% sodium azide prior to imaging ChR2-eYFP+ neurons in the cerebral cortex with the probe [29].

Supplementary 4- Calculation of the variation of length of probe and FOV

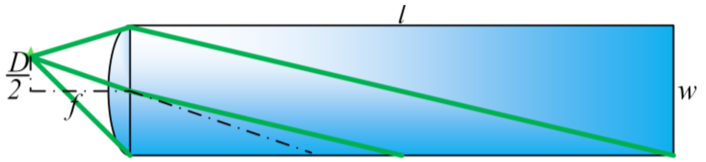

Supplementary Figure 4- Fluorescence imaging of a microsphere using the endoscopic needle. (b) Side view of the fluorescence dye when excited via the probe.

The field of view in the probe is dependent on the focal distance (*f*) and length of the probe (*l*). Considering a perfect lens in front of the waveguide (Supplementary Fig.4) and using ray matrices calculations Supplementary [1], the relation between ray properties at focal plane (r_{in}, r'_{in}) and inside the waveguide (r_{out}, r'_{out}) is,

$$\begin{Bmatrix} r_{out} \\ r'_{out} \end{Bmatrix} = \begin{bmatrix} 1 & l \\ 0 & 1 \end{bmatrix} \begin{bmatrix} 1 & 0 \\ 0 & 1/n \end{bmatrix} \begin{bmatrix} 1 & 0 \\ -1/f & 1 \end{bmatrix} \begin{bmatrix} 1 & f \\ 0 & 1 \end{bmatrix} \begin{Bmatrix} r_{in} \\ r'_{in} \end{Bmatrix},$$

where *n* is the waveguide refractive index. The microlens collimates the light from each point source located at the focal plane of the microlens in different angle and couple it to the waveguide. In order to be able to construct the image of the specific point source, at least part of the collimated beam needs to be collected by the objective lens before it changes its angle by hitting the waveguide wall. The marginal ray coming from a point source located on focal plane at *D/2* (half of the field of view diameter) hits the waveguide wall at *–w/2*. Using the above equation length of waveguide would be defined as *l=2nfw/D*. In the above calculation, the waveguide is considered as a circular (symmetric), in the case of the square waveguides, the waveguide width should be replaced by $w_{eq} = \sqrt{\frac{4}{\pi}}w$.

Supplementary References

[1] Yariv, A., & Yeh, P. (2006). Photonics: optical electronics in modern communications. Oxford University, ch.2, pp 66-106.
